# Steam distillation/drop-by-drop extraction with gas chromatography–mass spectrometry for fast determination of volatile components in jujube (*Ziziphus jujuba* Mill.) extract

**DOI:** 10.1186/s13065-017-0329-6

**Published:** 2017-10-13

**Authors:** Shi-Hao Sun, Guo-Bi Chai, Peng Li, Jian-Ping Xie, Yue Su

**Affiliations:** 1 Center for Chinese Medicine Therapy and Systems Biology, Shanghai University of Traditional Chinese Medicine, Shanghai, 201203 Shanghai China; 2 Key Laboratory in Flavor & Fragrance Basic Research, Zhengzhou Tobacco Research Institute, China National Tobacco Corporation, Zhengzhou, 450001 China

**Keywords:** Steam distillation, Drop-by-drop extraction, Volatile components, GC–MS, Jujube (*Ziziphus jujuba* Mill.) extract

## Abstract

**Background:**

Jujube extract is commonly used as a food additive and flavoring. The unique jujube aroma and the mild sweet aroma of the extract are critical factors that determine product quality and affect consumer acceptability. The aroma changes with changes in the extraction condition, which is typically dependent on the characteristics of volatile oils in the extract. Despite their importance, the volatile oils of jujube extract have received less attention compared with the soluble components. So, an appropriate qualitative and quantitative method for determination of the volatile oils is vitally important for quality control of the product.

**Results:**

A method coupling steam distillation/drop-by-drop extraction with gas chromatography–mass spectrometry (S3DE/GC–MS) was developed to determine the volatile components of jujube extract. Steam distillation was coupled with solvent extraction; the resulting condensate containing volatile components from jujube extract was drop-by-drop extracted using 2 mL of methyl tertiary butyl ether. The solvent served two purposes. First, the solvent extracted the volatile components from the condensate. Second, the volatile components were pre-concentrated by drop-by-drop accumulation in the solvent. As a result, the extraction, separation, and concentration of analytes in the sample were simultaneously completed in one step. The main parameters affecting the S3DE procedure, such as the water steam bubbling rate, extraction solvent volume, sample weight and S3DE time, were optimized. The standard addition approach was essential to obtain accurate measurements by minimizing matrix effects. Good linearity (R^2^ ≥ 0.9887) and good repeatability (RSDs ≤ 10.35%, n = 5) for 16 analytes in spiked standard analyte samples were achieved.

**Conclusions:**

With the S3DE/GC–MS method, seventy-six volatile compounds from jujube extract were identified and the content of 16 compounds was measured. The results were similar to those from simultaneous distillation extraction. The developed method was simple, fast, effective, sensitive, and provided an overall profile of the volatile components in jujube extract. Thus, this method can be used to determine the volatile components of extracts.

**Graphical abstract:**
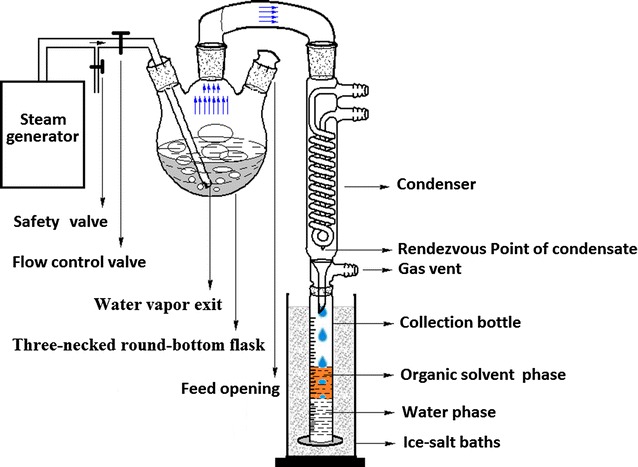
The diagram of steam distillation/drop-by-drop extraction device.

**Electronic supplementary material:**

The online version of this article (doi:10.1186/s13065-017-0329-6) contains supplementary material, which is available to authorized users.

## Introduction

Jujube (*Ziziphus jujuba* Mill.) is widely distributed in subtropical areas of the northern hemisphere, especially in China [1]. It has been commonly used in functional foodstuffs and crude drugs in traditional Chinese medicine [2, 3]. Jujube extract is usually used as a food additive or flavoring and is listed in the “lists of food additives” in China [4].

Jujube extract is a reddish-brown, semi-liquid substance obtained by extracting jujube fruits using different concentration of ethanol in water. The unique jujube aroma and the mild sweet aroma of the extract are critical factors that determine product quality and affect consumer acceptability [5]. The aroma changes with changes in the extraction condition, which is typically dependent on the characteristics of volatile oils in the extract. Despite their importance, the volatile oils of jujube extract have received less attention compared with the soluble components [6–8].

Gas chromatography–mass spectrometry (GC–MS) is typically employed to analyze volatile components in flavorings. Prior to GC–MS analysis, volatile components were isolated from nonvolatile mixtures, which required sample preparation steps to transfer the analyte into a pre-purified and concentrated form compatible with the analytical system [9]. Commonly used methods for isolating volatile components from natural sources include thermal desorption or vapor collection by cryogenic concentration or by adsorption on solid adsorbents, direct solvent extraction (e.g., Soxhlet and liquid–liquid extraction) [10, 11].

Thermal desorption and vapor collection are unreproducible and prone to artifacts, especially when working in the ppm range [12]. The advantages of direct solvent extraction are that most volatile compounds (low, medium, and high volatility) can be separated in one step, and good analytical precision can be achieved. However, direct extraction with a solvent co-solubilizes non-volatile components, which may contaminate the injectors and limit the analyte concentration [13]. Furthermore, large volumes of organic solvent, long extraction times, and concentration steps are required. Finally, compounds with low boiling points may be entirely missing in the solvent evaporation step.

In recent years, simple, rapid techniques that are solvent-free or require only small amounts of solvent, such as supercritical fluid extraction [14], headspace solid-phase microextraction [15–17], headspace liquid-phase microextraction (HS-LPME) [18, 19], and stir-bar sorptive extraction [20], have been widely used to characterize the volatile components of complex matrices. However, these methods often had poor precision. Recently, a method coupling hydro-distillation with static HS-LPME was developed and applied to determine the essential oil components of a natural material; this was a fast, low-cost, facile and efficient method [9, 21]. Despite a poor repeatability, e.g., between 17 and 19% for main components and even worse for minor components, this HS-LPME method provides a good basis for developing a more effective method.

Steam distillation is a popular approach to obtain volatile oils from natural materials. However, it has rarely been employed for the analysis of volatile oils in natural extracts. Small sample amounts are often used in analytical experiments, resulting in fractions of volatile oils too low to be effectively separated. In 1964, Likens et al. [22] introduced simultaneous distillation extraction (SDE) by combining steam distillation and extraction. However, extracts obtained by SDE must be concentrated to reach the minimal sensitivity required for GC.

Godefroot et al. [12] further improved SDE to enable determination following 2 h extractions using a microapparatus and without requiring any concentration steps before gas chromatography. In 1983, Bicchi et al. [23] made improvements to the microapparatus to decrease the volume of solvent used to 100 μL and to avoid hot organic solvent reflux. Bicchi et al. also standardized the operating conditions of the apparatus. More recently, Wei et al. [24] improved the microapparatus by simplifying the operating conditions and isolating volatile oils in natural materials. However, volatile components with low boiling points may be lost. Although the microapparatus is commercially available and has been used for extracting volatile components from natural materials, few practical applications have been reported for accurate quantitative analyses. Currently, methods that couple SDE with concentration steps are popular approaches for analyzing volatile components isolated from matrices. However, long extraction times (> 2 h) and large volumes of organic solvents (> 50 mL) are required [25–27]. Similar to direct solvent extraction, the concentration step after SDE may exclude compounds with low boiling points.

This work presents a new sample preparation method, steam distillation/drop-by-drop extraction (S3DE), to effectively extract, separate, and pre-concentrate volatile constituents in extracts. We also developed an easy-to-use approach to isolate and quantitatively analyze volatile components in jujube extracts with minimal solvent volumes at room temperature in a reasonable time. A comparison study with SDE was also carried out to benchmark the performance of the new approach.

## Experimental

### Material and reagents

Jujube extract was purchased from Zhengzhou Jieshi chemical company, China. The extract was produced by the following procedure. The jujube (*Ziziphus jujuba* Mill.) fruit was cleaned and denucleated. The pitted jujubes were then crumbed and extracted using 65% alcohol for 2 h at 70 °C. Then, the solvent was removed to produce the jujube extract.

Butanol, 3-methyl-1-butanol, 1-hexanol, 1-pentanol, 1-heptanol, 1-octanol, 1-nonanol, acetic acid, isobutyric acid, butyric acid, pentanoic acid, heptanoic acid, octanoic acid, capric acid, undecanoic acid, dodecanoic acid, 2-ethyl hexanol, furfural, 2-acetylfuran, benzaldehyde, 5-methylfurfural, 2-furanmethanol, dl-menthol, phenethyl alcohol, damascenone; ethyl hexanoate, ethyl heptanoate, ethyl caprylate, ethyl nonanoate, methyl caprate, ethyl caprate, diethyl succinate, methyl phenylacetate, ethyl phenylacetate, methyl laurate, phenethyl acetate, ethyl laurate, ethyl 3-phenylpropionate, methyl tetradecanoate, ethyl tetradecanoate, ethyl pentadecanoate, methyl hexadecanoate, ethyl hexadecanoate, ethyl heptadecanoate, ethyl stearate, ethyl oleate, ethyl linoleate, and styralyl propionate (as an internal standard) were purchased from J&K Scientific Ltd. Dichloromethane (chromatography grade) and methyl tertiary butyl ether (MTBE; chromatography grade) was provided by CNW technologies GmbH.

A mixed standard solution was prepared by resolving the chemicals in MTBE, including 3-methyl-1-butanol (3.53 mg/mL), 1-hexanol (0.29 mg/mL), furfural (0.63 mg/mL), ethyl caprate (0.38 mg/mL), menthol (0.26 mg/mL), 2-furanmethanol (0.26 mg/mL), ethyl phenylacetate (0.55 mg/mL), ethyl laurate (2.86 mg/mL), ethyl 3-phenylpropionate (0.25 mg/mL), phenylethyl alcohol (1.04 mg/mL), heptanoic acid (0.19 mg/mL), ethyl myristate (0.97 mg/mL), octanoic acid (0.32 mg/mL), ethyl hexadecanoate (2.21 mg/mL), decanoic acid (2.05 mg/mL), dodecanoic acid (12.71 mg/mL), ethyl oleate (0.91 mg/mL), and ethyl linoleate (0.23 mg/mL). An internal standard solution (3.58 mg/mL) was prepared by resolving styralyl propionate in MTBE.

### Instrumentation and steam distillation/drop-by-drop extraction procedure

**Fig. 1:**
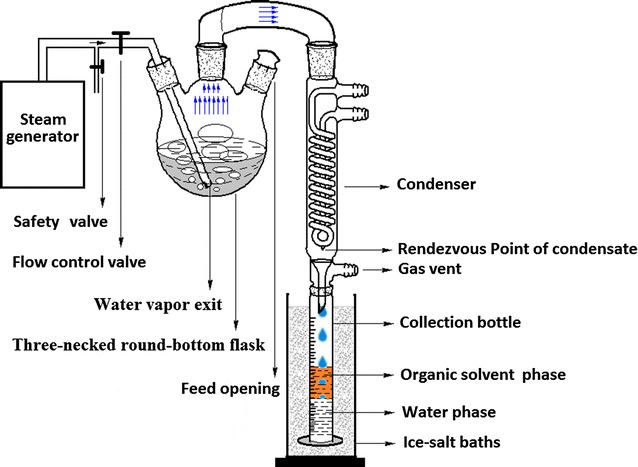
The diagram of steam distillation/drop-by-drop extraction device. (The device is suitable for extraction of volatile oils from extract. e.g. The jujube extract is produced by the following procedure: The jujube fruit was cleaned and denucleated. The pitted jujubes were then crumbed and extracted using alcohol. Then, the solvent was removed to produce the jujube extract)

A diagram of the S3DE apparatus is shown in Fig. 1. The apparatus primarily consists of a three-necked, round-bottom flask, a condenser, and a collection bottle. The S3DE procedure was as follows. First, the apparatus was assembled following the diagram shown in Fig. 1. Then, the condenser was switched to forced water circulation, which was cooled to 2–3 °C by a refrigeration system. After passing condensate water continuously through the condenser, a 3 g mixture of jujube extract and 20 mL of water were added into the three-necked, round-bottom flask. The water vapor exit was submerged in the mixture. Then, 2 mL of MTBE were spiked into the collection bottle, which was immersed into an ice-salt bath. A safety valve was closed, and water steam generated by a precise steam generator (flow > 10 g/min, 100–400 °C, approximately 0.5 MPa pressure; Suzhou Aros environment generator Co., Ltd.) was bubbled into the mixture. The vapor containing the volatile constituent of jujube extract flowed over into the condenser and was condensed as a liquid. This liquid was collected drop by drop into the collection bottle and was extracted by MTBE. The safety valve was opened, and the bottom bottle was removed after a determined extraction time. This MTBE solution was directly analyzed by GC–MS.

A quantitative comparison experiment was performed using SDE/GC–MS. SDE was conducted as described by Wang et al. [5]. Jujube extract (3 g) and 250 mL distilled water were mixed in a 1000-mL flask, and 60 mL dichloromethane was used as extraction solvent in a 100-mL flask. The two flasks were maintained at 120 and 60 °C by an electric jacket and a water bath, respectively. Each extraction was carried out for 3 h after the two arms started to reflux. After extraction, the dichloromethane extract was dried over anhydrous sodium sulfate overnight, concentrated to 2 mL and filtered through a 0.45-μm micropore film prior to GC–MS analysis.

### Gas chromatography/mass spectrometry

GC–MS analysis was performed using an Agilent 7890A gas chromatograph equipped with a DB-WAXetr capillary column (60 m × 0.25 mm, 0.25-μm coating thickness) and an Agilent 5975C mass detector. The analysis conditions were as follows: injector and transfer line temperature 250 and 280 °C, respectively; oven temperature increased from 50 °C (for 1 min) to 240 °C at 5 °C/min and was held at 240 °C for 10 min; helium carrier gas at 1 mL/min; 1 μL injection volume; and splitless. All samples for qualitative analyses were analyzed in full scan mode with a mass range of 33–500 amu. Selected ion monitoring (SIM) mode was used for quantitative analyses, the confirmative ions and the quantitative ions of the compounds are shown in Table 1.

**Table 1:** Retention time, linear retention index, area normalization percent content of the volatile components in jujube extract identified by the S3DE/GC–MS and confirmative ion and quantitative ion of the selected compound for quantitative analysis

No.	RT	Compounds	Area normalization percent content (%)	Identification method	LRI	Confirmative ion	Quantitative ion
1	9.887	2-Methyl-1-propanol	3.17	MS	–	–	–
2	11.148	1-Butanol	0.25	RI, MS, ST	1144	–	–
3	12.792	3-Methyl-1-butanol	8.45	RI, MS, ST	1207	70, 55	55
4	13.748	Ethyl capronate	0.07	RI, MS, ST	1243	–	–
5	13.987	1-Pentanol	0.14	RI, MS, ST	1252	–	–
6	15.818	4-Methyl-2-hexanol	0.04	RI, MS	1321	–	–
7	16.372	Ethyl heptanoate	0.06	RI, MS, ST	1341	–	–
8	16.705	1-Hexanol	0.54	RI, MS, ST	1354	84, 69	69
9	17.204	1,2-Dimethyl-cyclopent-2-enecarboxylic acid	0.12	RI, MS	1372	–	–
10	19.046	Ethyl caprylate	0.07	RI, MS, ST	1443	–	–
11	19.371	1-Heptanol	0.28	RI, MS, ST	1455	–	–
12	19.452	Acetic acid	0.13	RI, MS, ST	1458	–	–
13	20.087	Furfural	0.55	RI, MS, ST	1483	96, 95	96
14	20.305	2-Ethyl-1-hexanol	0.08	RI, MS, ST	1491	–	–
15	20.409	Ethyl 7-octenoate	0.06	RI, MS	1495	–	–
16	21.169	1-(2-Furanyl)-ethanone	0.13	RI, MS, ST	1526	–	–
17	21.527	Ethyl nonanoate	0.02	RI, MS, ST	1540	–	–
18	21.648	Propanoic acid	0.06	RI, MS	1545	–	–
19	21.849	Benzaldehyde	0.12	RI, MS, ST	1553	–	–
20	22.022	1-Octanol	0.22	RI, MS, ST	1560	–	–
21	22.367	2-Methyl-propanoic acid	0.05	RI, MS, ST	1574	–	–
22	22.917	5-Methyl-2-furan-carboxaldehyde	0.17	RI, MS, ST	1596	–	–
23	23.021	Hexadecane	0.02	RI, MS, ST	1600	–	–
24	23.125	Methyl caprate	0.05	RI, MS, ST	1604	–	–
25	23.845	Butanoic acid	0.06	RI, MS, ST	1635	–	–
26	24.124	Ethyl caprate	0.39	RI, MS, ST	1646	155, 101	101
27	24.239	Menthol	0.40	RI, MS, ST	1651	138, 128	128
28	24.359	1-Nonanol	0.08	RI, MS, ST	1656	–	–
29	24.676	2-Furanmethanol	0.30	RI, MS, ST	1670	98, 81	98
30	25.121	Diethyl succinate	0.02	RI, MS, ST	1688	–	–
31	26.082	5-Methyl-2-Furanmethanol	0.04	RI, MS	1730	–	–
32	26.394	Pentanoic acid	0.10	RI, MS, ST	1744	–	–
33	26.859	1,2-Dimethyl-4-oxocyclohex-2-enecarboxaldehyde	0.03	RI, MS	1765	–	–
34	26.921	1-Decanol	0.02	RI, MS	1767	–	–
35	27.153	Naphthalene	0.02	RI, MS	1778	–	–
36	27.248	Methyl phenylacetate	0.07	RI, MS, ST	1782	–	–
37	27.808	Ethyl phenylacetate	0.39	RI, MS, ST	1807	164, 91	91
38	27.901	Methyl laurate	0.24	RI, MS, ST	1811	–	–
39	28.505	Phenethyl acetate	0.40	RI, MS, ST	1839	–	–
40	28.672	Damascenone	0.14	RI, MS, ST	1847	–	–
41	28.765	Ethyl laurate	4.03	RI, MS, ST	1851	183, 101	101
42	29.639	1-Methyl-naphthalene	0.09	RI, MS	1891	–	–
43	30.003	Ethyl 3-phenylpropionate	0.33	RI, MS, ST	1909	178, 104	104
44	30.493	Phenylethyl alcohol	0.71	RI, MS, ST	1932	122, 91	91
45	30.919	5-Butyldihydro-2(3H)-furanone	0.08	RI, MS	1953	–	–
46	31.013	Heptanoic acid	0.38	RI, MS, ST	1958	87, 73	73
47	31.2	Isobutyl laurate	0.08	RI, MS	1967	–	–
48	32.001	4-hydroxy-4-methyl-4H-naphthalen-1-one	0.15	RI, MS	2006	–	–
49	32.251	Methyl myristate	0.05	RI, MS, ST	2019	–	–
50	33.000	Ethyl myristate	2.68	RI, MS, ST	2057	101, 88	88
51	33.156	Octanoic acid	0.79	RI, MS, ST	2065	73, 60	73
52	33.364	Isoamyl laurate	0.06	RI, MS	2075	–	–
53	33.666	Ethyl tetradecenoate (I)	0.09	RI, MS	2090	–	–
54	33.791	Ethyl tetradecenoate (II)	2.43	RI, MS	2097	–	–
55	33.947	Ethyl tetradecenoate (III)	0.02	RI, MS	2105	–	–
56	34.54	6,10,14-Trimethyl-2-pentadecanone	0.19	RI, MS	2136	–	–
57	34.967	Ethyl pentadecanoate	0.13	RI, MS, ST	2159	–	–
58	35.217	Nonanoic acid	0.25	RI, MS, ST	2172	–	–
59	36.216	Methyl hexadecanoate	0.27	RI, MS, ST	2226	–	–
60	36.882	Ethyl hexadecanoate	6.32	RI, MS, ST	2263	101, 73	101
61	37.006	Methyl (Z)-9-hexadecenoate	0.12	RI, MS	2270	–	–
62	37.131	Decanoic acid	5.08	RI, MS, ST	2276	129, 73	73
63	37.412	Ethyl hexadecenoate (I)	3.50	RI, MS	2292	–	–
64	37.652	Ethyl hexadecenoate (II)	0.13	RI, MS	2305	–	–
65	37.724	Ethyl hexadecenoate (III)	5.06	RI, MS	2309	–	–
66	38.13	Dimethyl phthalate	0.25	RI, MS	2333	–	–
67	38.682	Ethyl heptadecanoate	0.07	RI, MS, ST	2364	–	–
68	39.054	Undecanoic acid	0.17	RI, MS, ST	2386	–	–
69	40.524	Ethyl octadecanoate	0.23	RI, MS, ST	2466	–	–
70	40.94	Dodecanoic acid	27.04	RI, MS, ST	2489	200, 171	200
71	41.398	Ethyl oleate	0.30	RI, MS, ST	2512	264, 222	264
72	41.98	Ethyl linoleate	0.35	RI, MS, ST	2540	109, 95	109
73	42.542	Isobutyl phthalate	2.90	RI, MS	2566	–	–
74	45.716	Tetradecanoic acid	7.64	RI, MS	2698	–	–
75	46.382	Dibutyl phthalate	2.91	RI, MS	2721	–	–
76	47.173	Z-7-Tetradecenoic acid	7.57	RI, MS	2747	–	–

### Identification of volatile components in jujube extract

The volatile components in jujube extract were identified using the NIST11 and Wiley databases and retention indices. Linear retention indices were obtained using gas chromatograms by interpolation between bracketing n-alkanes [28–30]. A homologous series of n-alkanes (C-7 to C-40; ULTRA Scientific, Inc.; North Kingstown, USA) was used as a standard. A few targets were further confirmed using standard compounds.

### Quantitative analysis of volatile components in the jujube extract

The quantitative analyses of volatile components in the jujube extract were performed using the standard addition approach. All data presented in this paper are averages of five replicates unless otherwise stated. Calibration curves were constructed by determining the peak area ratio of analytes-to-internal standard (Y) versus the amount of spiked standard analytes (X). Method precision was evaluated using relative standard deviation (RSD), and recovery rates were measured following the procedure of Wu et al. [18, 31]. Analyte recovery (five replicate tests) was calculated as (mean calculated amount/nominal amount) × 100%.

## Results and discussion

### Steam distillation/drop-by-drop extraction and GC–MS analysis

Steam distillation is a good method to obtain volatile oils from large amounts of plant materials. When vapor-capturing volatile oils are sufficiently cooled, the oil naturally separates from the hydrosol [9]. A small amount of the oil is often used for instrument analysis. However, the obtained volatile oils are typically at trace levels too difficult to effectively separate.

In this study, volatile components in jujube extract were extracted by the device shown in Fig. 1. This S3DE extraction process is based on the basic principles of steam distillation and extraction. As water steam is continuously bubbled into a jujube extract solution in the three-necked, round-bottom flask, the vapor captures the volatile components of the jujube extract. The vapor is then transferred under pressure and cooled in the condenser. As the vapor cools, liquid condensate drops, containing the volatile components, are formed and collected in a collection bottle. (The drop formation rate of the liquid condensate can be controlled by modifying the water steam bubbling rate). When an organic solvent less dense than water is present in the collection bottle, the condensate drop can naturally pass through the solvent layer and gather at the bottom of the collection bottle. The volatile components in the drops are extracted into the organic solvent as the drop passes through the organic layer. Thus, the volatile components of the jujube extract can be extracted into the organic phase.

The extraction solvent should be carefully selected to achieve the desired extraction. In this study, MTBE, an organic solvent with a density less than that of water, was used as the extraction solvent and spiked into the collection bottle to extract the condensate without optimization.

**Fig. 2:**
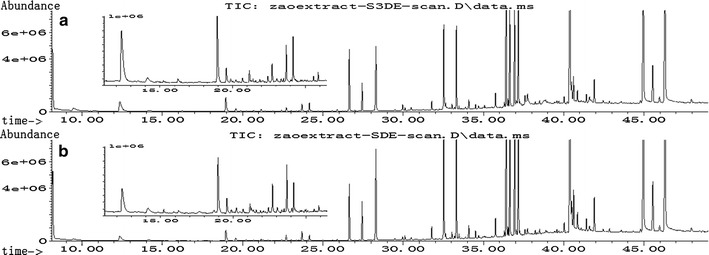
The GC/MS chromatogram of volatile components in jujube extract. The samples of **a** and **b** were prepared by S3DE and SDE, respectively

Volatile oils naturally separate from hydrosols. As the water steam vapor is condensed, the volatile oils continuously separate from the hydrosol. As a result, the volatile oils are present on the surfaces of the forming drops. When the drops enter the organic solvent layer in the collection bottle, the surface-dwelling volatile oils are desorbed into the organic solvent while the water phase drops pass through the solvent layer. As these aqueous drops are collected in the collection bottle, the volatile oils are concentrated in the organic solvent. This organic solvent phase can then be directly analyzed by GC–MS, as is shown in the chromatogram in Fig. 2a.

The volatile components in the jujube extract were identified using the NIST11 and Wiley databases and the retention indices. Other analytes were also confirmed using standard compounds. The results are summarized in Table 1.

### Parameter optimization of S3DE

Various volatile components with different boiling points, including 3-methyl-1-butanol, 1-heptanol, ethyl caprate, ethyl laurate, ethyl hexadecanoate, and dodecanoic acid, are present in jujube extract and were selected as targets to optimize the extraction parameters, such as the water steam bubbling rate, MTBE volume, sample weight and S3DE time. After the extraction was completed, the MTBE solution containing the analytes was directly injected into the GC/MS system for analysis. All quantifications were based on the relative peak area of the analytes to the internal standards unless otherwise stated.

#### Bubbling rate of water steam

The water steam bubbling rate is a key factor that affects the efficiency of steam-distillation. A higher bubbling rate typically provides better distillation efficiency. However, if the bubbling rate is too high, the vapor with volatile components would not be completely cooled by the condenser. Furthermore, the condensate would be generated so fast it would be impossible to achieve a drop-by-drop extraction procedure. In this study, we modified the water steam bubbling rate using a control valve to adjust the condenser efficiency. As a result, the condensates were drop-by-drop collected into the collection bottle at a rate of 2 drops/1 s.

#### Volume of MTBE

**Fig. 3:**
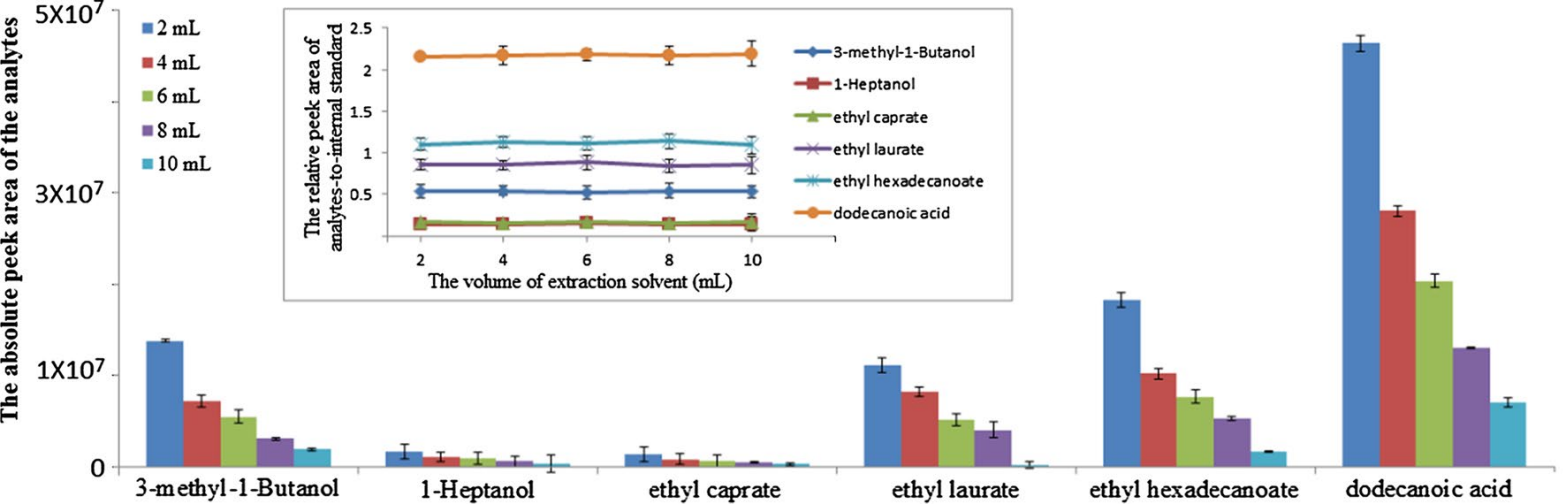
Optimization of the extraction solvent volume

Preliminary experiments were performed to optimize the volume of MTBE. The results (Fig. 3) indicated that the relative peak area of the analytes-to-internal standard did not significantly change, whereas the absolute peak area of the analytes decreased with increasing MTBE volume within a set S3DE time. Thus, smaller volumes of MTBE should be used. In practice, the solvent volume typically decreases with increasing S3DE time due to solvent volatility. For convenience-sake, a 2-mL volume of solvent, ideal for GC–MS automatic injection, was used in the S3DE experiments. After S3DE, 1 mL of the MTBE solvent with volatile components was further analyzed using GC–MS.

#### Weight of sample

A number of studies have confirmed that the weight of the sample is dependent on the requirements of the analytical instrument. Preliminary experiments showed that the absolute peak area of the selected analytes increased with increasing sample weight. To explore the influence of sample weight on the extraction efficiency of the volatile components in jujube extract, the sample weight was optimized over a 1–10 g range (data not shown). When 1 g of jujube extract was used, a long S3DE time was required to extract sufficient amounts of low content volatile compounds to meet GC–MS minimum detection limit requirements. However, for high content volatile compounds, a prolonged S3DE would result in over-extraction, which may overload the chromatographic column. As a compromise, 3 g sample weights were used.

#### S3DE time

In general, the amount of volatile components extracted from sample increases with steam-distillation time. During S3DE, solvent extraction was performed following steam-distillation. Experimental results showed that the drop-by-drop extraction and steam-distillation were nearly simultaneous after the first drop of condensate formed in the condenser. Thus, the efficiency of solvent extraction and steam-distillation is primarily dependent on the steam-distillation time, or “S3DE time”. The S3DE time is defined as the time from the formation of the first drop of condensate in the condenser to the time at which the collection bottle is removed.

**Fig. 4:**
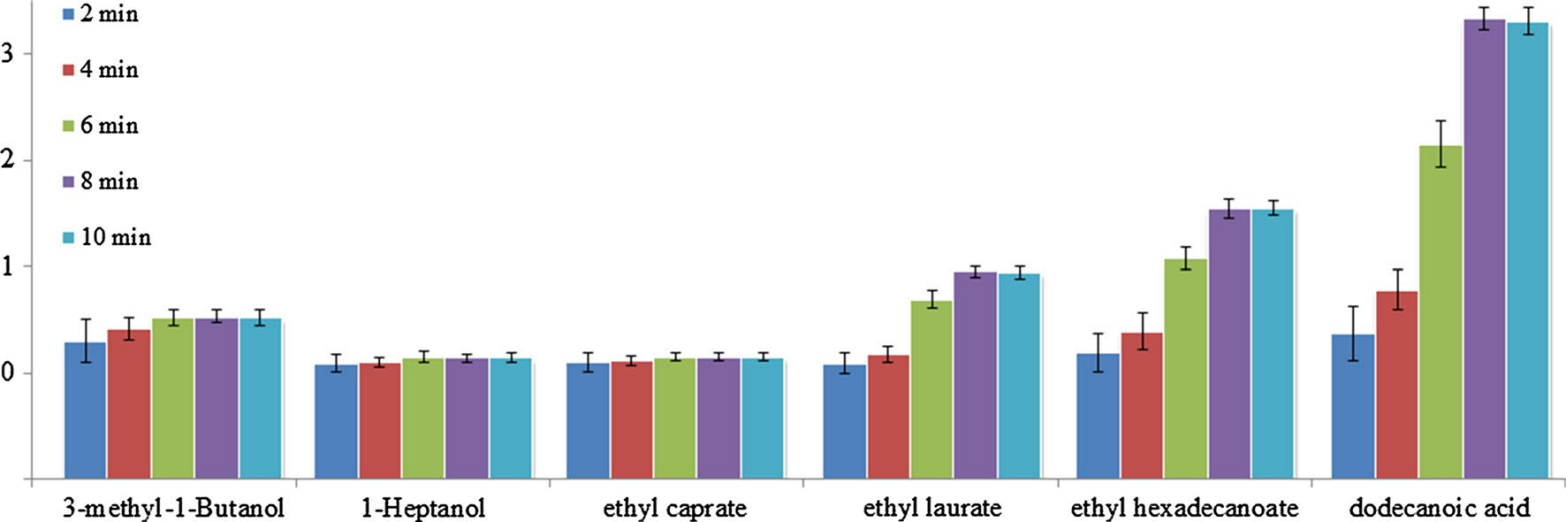
Optimization of the S3DE time

A series of experiments were performed to optimize the S3DE time (i.e., 2, 4, 6, 8, and 10 min), as shown in Fig. 4. The amount of analytes extracted by S3DE was dependent on the S3DE time. The GC–MS data showed that the absolute peak area of all analytes increased with increasing S3DE time. The results also showed that the relative peak area of the analytes-to-internal standard was roughly constant when the S3DE time was at least 8 min. Thus, 8 min was selected as the S3DE time for further experiments.

### Validation of S3DE-GC/MS method

An analytical method should not be influenced by the sample matrix. A blank matrix is always desired for all types of quantitative analyses. However, a blank matrix is usually not available, especially for natural samples. The standard addition approach may be a good alternative way to quantitatively analyze a sample and can compensate for differences in sample matrices [18, 32–35]. This approach makes use of the addition of known amounts of analytes of interest to multiple aliquots of the sample and of another non-spiked, baseline aliquot, i.e., the “zero-point”. Then, after the samples are analyzed, a calibration curve of the measured values is plotted against the spiked amounts for each sample aliquot. A straight line is drawn and the value of the X intercept represents the amount of analyte in the unknown sample [18, 31, 36, 37].

In this study, 18 volatile compounds in the extract were selected to validate the S3DE-GC/MS method. An ion monitor was employed for the mass spectrometry analysis of the analytes to identify and measure the level of ions as summarized in Table 1. A series of amounts (0, 20, 40, 60, and 120 μL) of standard solution were spiked into the three-necked, round-bottom flask containing 3 g jujube extract with an internal standard. The samples were then analyzed by the developed method. The calibration curve of each target analyte was constructed and is shown in Table 2.

**Table 2:** Calibration curves of 18 target analytes

Name	Calibration curves	R^2^	LOD (μg/g)	Recovery	
				Value (%)	RSD(%)
3-Methyl-1-butanol	Y = 0.0089X + 0.8730	0.9987	3.16	91.33	10.10
1-Hexanol	Y = 0.0039X + 0.01150	0.9931	0.15	95.62	9.81
Furfural	Y = 0.0062X + 0.0463	0.7099	0.97	74.19	27.44
Ethyl caprate	Y = 0.0066X + 0.0398	0.9990	1.02	103.68	8.24
Menthol	Y = 0.0035X + 0.0239	0.9939	0.61	97.38	8.35
2-Furanmethanol	Y = 0.0016X + 0.0104	0.8042	1.16	79.22	19.03
Ethyl phenylacetate	Y = 0.0300X + 0.2147	0.9991	0.11	96.33	5.41
Ethyl laurate	Y = 0.0085X + 0.4157	0.9962	1.86	99.21	5.87
Ethyl 3-phenylpropionate	Y = 0.0168X + 0.0494	0.9992	0.41	98.45	8.25
Phenylethyl alcohol	Y = 0.0075X + 0.1132	0.9993	1.03	97.61	6.15
Heptanoic acid	Y = 0.0029X + 0.0201	0.9887	1.15	87.06	11.06
Ethyl myristate	Y = 0.0218X + 0.6532	0.9971	2.67	97.99	3.52
Octanoic acid	Y = 0.0024X + 0.0278	0.9895	1.65	90.16	9.17
Ethyl hexadecanoate	Y = 0.0143X + 0.9181	0.9979	3.41	100.04	5.28
Decanoic acid	Y = 0.0096X + 0.4570	0.9894	3.94	93.54	8.66
Dodecanoic acid	Y = 0.0913X + 30.027	0.9981	4.15	95.59	7.94
Ethyl oleate	Y = 0.0028X + 0.0390	0.9990	0.84	94.80	8.36
Ethyl linoleate	Y = 0.0026X + 0.0183	0.9980	0.75	92.34	10.95

A few performance parameters, including linearity, limits of detection (LODs), repeatability and recovery, were investigated using samples with unknown levels of volatile components. A linear response was observed for the added standard stock solutions from 0 to 120 μL with a high coefficient of determination (R^2^ ≥ 0.9821), excluding furfural (R^2^ = 0.7084), 2-furanmethanol (R^2^ = 0.8051) and heptanoic acid (R^2^ = 0.9087). The relative standard deviation (RSD) was less than 13.97% and is shown in Table 3. Good LODs ranging from 0.11–4.15 μg/g were obtained, as based on three times the standard deviations from ten replicate tests at the “zero-point”. The recoveries of analytes were measured by spiking 20 μL of standard stock solution into the jujube extract sample, which was then analyzed as an unknown level sample. The results (shown in Table 2) were satisfactory except for furfural (74.19%, RSD = 27.44%, n = 5), 2-furanmethanol (79.22%,RSD = 19.03%, n = 5) and heptanoic acid (87.06%, RSD = 11.06%, n = 5). These excluded compounds had low recovery levels and poor linearity. These compounds likely had relatively large water solubility levels.

**Table 3:** Concentrations of volatile compound in jujube extract obtained by the S3DE method and the SDE method

Name	Concentration (μg/g)			
	S3DE method		SDE method	
	Value	Repeatability (RSD, %)	Value	Repeatability (RSD, %)
3-Methyl-1-Butanol	32.70	4.72	28.92	5.11
1-Hexanol	0.98	6.18	0.68	3.07
Ethyl caprate	2.01	5.11	1.67	2.25
Menthol	2.27	5.79	1.91	4.96
Ethyl phenylacetate	2.38	6.13	2.08	5.17
Ethyl laurate	16.3	4.83	16.3	4.13
Ethyl 3-phenylpropionate	0.98	5.91	0.81	2.39
Phenylethyl alcohol	5.10	4.25	4.59	4.23
Heptanoic acid	2.31	10.35	2.36	6.41
Ethyl myristate	9.99	4.81	10.37	3.17
Octanoic acid	3.86	6.87	3.66	5.68
Ethyl hexadecanoate	21.40	4.64	19.09	4.19
Decanoic acid	15.87	5.54	16.96	5.73
Dodecanoic acid	109.62	4.17	113.52	4.36
Ethyl oleate	4.64	6.24	5.77	5.27
Ethyl linoleate	2.35	6.31	1.75	6.48

### Quantitative analysis of volatile components in jujube extract

A jujube extract sample with unknown levels of volatile components was analyzed using the developed method. The levels of the volatile components in the sample were obtained by determining the X-intercept as shown in Table 3. The sample was also measured using a conventional SDE/GC–MS method. The chromatogram is shown in Fig. 2b, and the data relative to repeatability of the method (see Additional file 1 for more detail) are deposited in Table 3. Paired *t* test comparisons between the data collected by the S3DE method and the SDE method were performed using Microsoft Office Excel. The results indicated that there were no significant differences (*P* = *0.49*) between the yields of the sixteen components as determined by the two methods. However, a significant difference (*P* = *0.01*) was observed regarding repeatability. Although a better repeatability was obtained by the SDE method, the developed S3DE method required lower amounts of organic solvent and was a simpler, more rapid, and more accurate procedure for characterizing the volatile components in jujube extract. A review of our experimental procedure and a rigorous standardization of the operating conditions may be helpful to improve the repeatability of the S3DE method, which will be further investigated.

## Conclusions

A simple sample preparation procedure was developed to characterize the volatile components in jujube extract. In this procedure, condensates from steam-distillation were drop-by-drop extracted in a small volume of organic solvent. The extraction procedure was performed immediately after steam-distillation. As a result, the extraction, separation, and pre-concentration of analytes in the sample were simultaneously completed. This minimal-solvent approach proved to be a simple, rapid, and accurate procedure for the determination of volatile components in jujube extract. Good linearity (R^2^ ≥ 0.9887) and good repeatability (RSDs ≤ 6.87%, n = 5) were achieved for 16 analytes in a spiked standard sample, excluding heptanoic acid (RSD = 10.35%). This new approach can be used as an alternative in the analysis of volatile fractions in extracts and complex matrices and provides certain advantages, including simple operation and lower time, energy and organic solvent requirements.

## Additional file

**Additional file 1.** Data about the performance of the SDE method.
