# Supplementary material for: Steam distillation/drop-by-drop extraction with gas chromatography–mass spectrometry for fast determination of volatile components in jujube (Ziziphus jujuba Mill.) extract
Source: Chem Cent J. 2017 Oct 13;11:101. doi: 10.1186/s13065-017-0329-6 (PMC5640556; doi:10.1186/s13065-017-0329-6)
Supplement: Supplementary file 1 — Additional file 1. Data about the performance of the SDE method. [file 13065_2017_329_MOESM1_ESM.docx]

**Additional File**

The SDE/GC–MS method described by Wang, et al [Anal Lett 2014, 47:654.], with a little change of gas chromatographic conditions, was employed to perform the quantitative comparison experiment.

A series of working standard solutions of targets were prepared in the concentration range of 1-2500 μg/mL and analyzed by the SDE/GC–MS method. Calibration curves were constructed by plotting the peak area ratio of analyte-to-internal standard (Y) versus concentration of analyze (X). The standard solution with the lowest concentration of the calibration curves was analyzed for 10 times, and then their standard deviation (SD) was calculated. LOD and LOQ were defined, respectively, as three times of SD and ten times of SD. Recovery experiment was performed on the spiked jujube extract at three spiking levels. Analyte recovery was calculated as (mean calculated amount/nominal amount)×100%. The data are summarized as S-table 1 and S-table 2.

**S-Table S1 Calibration curves, LODs, and LOQs of 18 target analytes obtained by the SDE method**

| Compounds | Calibration curves | R^2^ | LODs (μg/mL) | LOQs(μg/mL) |
| --- | --- | --- | --- | --- |
| 3-methyl-1-Butanol | Y=0.7172X+0.0204 | 0.9991 | 0.28 | 0.93 |
| 1-Hexanol | Y=0.0231X-0.0347 | 0.9978 | 0.02 | 0.07 |
| furfural | Y=0.1113X+0.0032 | 0.9999 | 0.04 | 0.13 |
| Ethyl caprate | Y=0.0836X-0.1038 | 0.9997 | 0.01 | 0.03 |
| Menthol | Y=0.0805X-0.0140 | 0.9981 | 0.07 | 0.24 |
| 2-furanmethanol | Y=0.0827X-0.0219 | 0.9995 | 0.11 | 0.36 |
| Ethyl phenylacetate | Y=0.4896X-0.0410 | 0.9994 | 0.03 | 0.10 |
| Ethyl laurate | Y=0.1829X+0.0532 | 0.9988 | 0.14 | 0.47 |
| Ethyl 3-phenylpropionate | Y=0.0097X-0.0017 | 0.9983 | 0.03 | 0.10 |
| Phenylethyl alcohol | Y=0.0911X+0.0105 | 0.9998 | 0.09 | 0.30 |
| Heptanoic acid | Y=0.0438X-0.0013 | 0.9988 | 0.08 | 0.27 |
| Ethyl myristate | Y=0.1824X+0.0632 | 0.9993 | 0.21 | 0.71 |
| Octanoic acid | Y=0.0630X-0.0081 | 0.9992 | 0.10 | 0.34 |
| Ethyl hexadecanoate | Y=0.5635X-0.0136 | 0.9984 | 0.36 | 1.19 |
| Decanoic acid | Y=0.4946X+0.0017 | 0.9996 | 0.23 | 0.78 |
| Dodecanoic acid | Y=1.8160X+0.5412 | 0.9996 | 0.32 | 1.07 |
| Ethyl oleate | Y=0.3012X+0.0127 | 0.9986 | 0.10 | 0.32 |
| Ethyl linoleate | Y=0.0931X-0.0306 | 0.9995 | 0.11 | 0.36 |

**S-Table S2 Recoveries of 18 target analytes obtained by the SDE method**

| Compounds | Concentration (μg/g) in the jujube extract | Recovery (%) | | |
| --- | --- | --- | --- | --- |
|  |  | L | M | H |
| 3-methyl-1-Butanol | 28.92 | 82.12 | 80.28 | 85.37 |
| 1-Hexanol | 0.68 | 69.94 | 76.15 | 82.35 |
| Furfural | 3.31 | 75.66 | 80.78 | 94.5 |
| Ethyl caprate | 1.67 | 88.54 | 89.18 | 90.07 |
| Menthol | 1.91 | 80.47 | 81.76 | 83.98 |
| 2-furanmethanol | 1.98 | 77.19 | 81.45 | 83.62 |
| Ethyl phenylacetate | 2.08 | 80.25 | 86.26 | 90.15 |
| Ethyl laurate | 16.3 | 90.17 | 93.25 | 98.14 |
| Ethyl 3-phenylpropionate | 0.81 | 88.54 | 89.02 | 92.71 |
| Phenylethyl alcohol | 4.59 | 82.74 | 86.70 | 90.13 |
| Heptanoic acid | 2.36 | 90.42 | 90.25 | 92.11 |
| Ethyl myristate | 10.37 | 96.89 | 97.83 | 95.74 |
| Octanoic acid | 3.66 | 99.18 | 100.01 | 102.35 |
| Ethyl hexadecanoate | 19.09 | 98.46 | 98.67 | 93.17 |
| Decanoic acid | 16.96 | 97.17 | 99.37 | 105.15 |
| Dodecanoic acid | 113.52 | 95.16 | 90.24 | 89.41 |
| Ethyl oleate | 5.77 | 105.27 | 97.23 | 96.58 |
| Ethyl linoleate | 1.75 | 101.01 | 95.42 | 97.37 |

**L: the spiked recoveries of low spiking level (50%); M: the spiked recoveries of middle spiking level (100%); Re-H: the spiked recoveries of high spiking level (150%).**
